# Addressing non-communicable diseases in Malaysia: an integrative process of systems and community

**DOI:** 10.1186/1471-2458-14-S2-S4

**Published:** 2014-06-20

**Authors:** Feisul Idzwan Mustapha, Zainal Ariffin Omar, Omar Mihat, Kamaliah Md Noh, Noraryana Hassan, Rotina Abu Bakar, Azizah Abd Manan, Fatanah Ismail, Norli Abdul Jabbar, Yusmah Muhamad, Latifah A Rahman, Fatimah A Majid, Siti Nurbaya Shahrir, Eliana Ahmad, Tamzyn Davey, Pascale Allotey

**Affiliations:** 1 Disease Control Division, Ministry of Health, Putrajaya Malaysia, Level 2, Block E3, Complex E, 62590 Putrajaya, Malaysia; 2 Family Health Development Division, Ministry of Health, Putrajaya Malaysia; 3 Melaka State Health Department, Malaysia; 4 Negeri Sembilan State Health Department, Seremban, Malaysia; 5 Pulau Pinang State Health Department, Malaysia; 6 Selangor State Health Department, Malaysia; 7 Johor State Health Department Malaysia; 8 Kedah State Health Department, Malaysia; 9 Pahang State Health Department Malaysia; 10 Global Public Health, Monash University, Malaysia

## Abstract

The prevalence of non-communicable diseases (NCDs) and NCD risk factors in Malaysia have risen substantially in the last two decades. The Malaysian Ministry of Health responded by implementing, “The National Strategic Plan for Non-Communicable Diseases (NSP-NCD) 2010-2014”, and the “NCD Prevention 1Malaysia” (NCDP-1M) programme. This paper outlines the primary health system context in which the NCDP-1M is framed. We also discuss the role of community in facilitating the integration of this programme, and outline some of the key challenges in addressing the sustainability of the plan over the next few years. The paper thus provides an analysis of an integration of a programme that involved a multi-sectoral approach with the view to contributing to a broader discourse on the development of responsive health systems.

## Introduction

Non-Communicable diseases (NCDs) are a significant and growing global public health problem affecting all countries, regardless of income [1]. The Malaysian National Health and Morbidity Survey which monitors NCD risk factors, indicated a three-fold rise in the prevalence of obesity, from 4.4% in 1996 to 15.1% in 2011 for adults age 18 years and above [2]. This equates to approximately 2.5 million Malaysians who in 2011 met the criteria for obesity. It is not surprising therefore that the prevalence of Type 2 diabetes also increased from 11.6% in 2006, to 15.2% in 2011, which equates to approximately 2.6 million adults. Hypertension also remains high at 35.1%, which equates to 5.8 million adult Malaysians. An estimated 32.7%, or 6.2 million adult Malaysians, were diagnosed in 2011 with hypercholesterolaemia [2]. The burden of disease attributable to overweight or obesity is likely underestimated with the NHMS 2011 indicating a high proportion of undiagnosed diabetics and hypertensives; the survey estimated that for every one person diagnosed with diabetes, another remains undiagnosed, and for every two known hypertensive adults, three remain undiagnosed.

In view of the increasing burden of NCDs in Malaysia, the Ministry of Health (MOH) has responded by developing and implementing the, “National Strategic Plan for Non-Communicable Diseases (NSP-NCD) 2010-2014”[3]. The NSP-NCD was developed in line with the mandates of the World Health Organization (WHO), particularly with reference to the, “2008-2013 Action Plan for the Global Strategy for the Prevention and Control of NCDs” and the “Western Pacific Regional Action Plan for NCDs”. However, the plan needed to be placed within the context of the Malaysian National Health System which has evolved in various ways since its introduction following the Alma Ata Declaration on Primary Health Care [4]. The health care system is based on an ethos of universal health coverage, recognising the crucial role of the community. The Malaysian health system has proven successful in significantly improving health outcomes for Malaysians compared to most countries at similar stages of economic development. Therefore the introduction of the NCDs strategic plan needed to be integrated effectively, building on existing strengths.

The aim of this paper is to outline the health system context in which the Malaysia Strategic Plan for Non-Communicable Diseases – “NCD Prevention 1Malaysia” (NCDP-1M) is framed. We also discuss the role of community in facilitating the integration and outline some of the key challenges in addressing the sustainability of the plan over the next few years. The paper thus provides an analysis of an integration of a program that involved a multi-sectoral approach with the view to contributing to a broader discourse on the development of responsive health systems.

## Primary health care in Malaysia

Primary health care, as defined by the Alma Ata Declaration is, “essential health care based on practical, scientifically sound and socially acceptable methods and technology made universally accessible to individuals and families in the community through their full participation and at a cost that the community and country can afford…It is the first level of contact of individuals, the family and the community with the national health system, bringing health care as close as possible to where people live and work” [5]. The primary health care system in Malaysia is a two-tier system comprising health clinics with a comprehensive range of primary medical care services (*klinik kesihatan* or KK), and community clinics (*klinik desa* or KD) with services largely limited to maternal and child health. A variation on the KDs is the 1Malaysia clinic, situated in high density, low income urban and semi-urban areas. Each KK serves a population of approximately 15,000 to 20,000 persons, while each KD provides services for approximately 2,000 to 4,000 mothers and children [6]. There are 985 health clinics, 1,864 community clinics, and 109 1Malaysia clinics [7]. Remote areas are served by mobile health clinics via land, river and air. There are currently 184 mobile health teams serving a total remote rural population of just over half a million people.

Primary health care services in Sabah and Sarawak work with a stronger integration of community members. The Village Health Promoter programme in Sarawak was implemented in 1983 with the aim to mobilise communities to adopt healthy behaviours, through agents of change from within the community itself. The programme still operates across Sarawak, with 1,600 Village Health Promoters currently in place.

The Primary Health Care Volunteer programme began in Sabah in 1987 as an anti-malarial preventive activity. While Primary Health Care Volunteers continue to promote active community participation, partnership, and responsibility, their role has expanded to support general health preventive activities. The key to success of these programmes is strong community participation, a structured training programme, support in terms of coordination from the Ministry, multi-sector participation, as well as monitoring and evaluation.

The referral system connects primary health care facilities with the hospitals (at both district and State level) and specialist centres [4]. The health clinics and hospitals have common policies and operating procedures to facilitate rapid and efficient management of referred cases. Community clinics are able to refer to health clinics or directly to the hospitals, according to established patient-management protocols. This system has helped Malaysia achieve gains in health status which are comparable to that in countries with higher health expenditure.

The strength of the primary health care system in Malaysia has been in the close-to-client maternal and child health care services which have increased the coverage of skilled attendants even in the remote rural areas of the country. This served to critically reduce maternal morbidity and mortality rates, increase integrated management of childhood illness and reduce under-5 mortality; all health indicators that are a critical reflection of the state of health and development for low- and middle-income countries. The penetration of this primary health care system has also facilitated the provision of acute care of communicable diseases and minor ailments. The increasing burden of NCDs, however, has resulted in the need for and focus on chronic care, and such demand has challenged the system given resources were originally designed for acute care [8]. In order to address the problem of NCDs, further evolution of the health care system was required to account for: a) the long-term needs of patients; and b) integration of various health clinic activities, like prevention and education, with treatment and follow-up. These strategies would necessarily involve inputs and participation at the community level.

A ‘Reviewed Approach’ to service delivery within the primary health care system, was adopted in 2007 [9]. The reviewed approach framework is represented by the acronym W.I.S.E., which denotes the various components of the services available at primary care clinics, namely: **W**ellness (health promotion, screening, and identification of risk factors); **I**llness (intervention and treatment); **S**upport services (rehabilitation and follow-up care); and **E**mergency services. Improved health informatics were also introduced to support the continuity of care across these services. This new approach required changes not only in staff capacity but also in the mix of specialities of staff required. Multi-disciplinary and multi-skilled primary health care teams were introduced, and clinics previously dedicated to specific conditions, had to adapt a more integrated approach. Currently, primary care clinics often include a mix of the following health professionals: family medicine specialists; general medical practitioners; physiotherapists; occupational therapists; nurses; assistant medical officers; nutritionists; and dieticians.

## Early integration of NCD management in primary care

The focus of NCD management within the primary health care system is on integrated and comprehensive service delivery at first point of contact. That is, primary care clinics are equipped to provide a full range of NCD services, including: promotive, preventive or wellness services; screening; identification of risk factors; intervention; treatment; and rehabilitation. All patients who present to primary health care facilities are offered screening services, irrespective of their entry point - outpatient, maternal and child health clinic, family planning services, or dental appointments. These preventive strategies were aimed at reducing exposure to risk via early risk identification (screening), risk intervention (education and health promotion), and risk management (monitoring of, for example, blood pressure, and body mass index).

Screening services in the primary care clinics were streamlined in 2007, with an integrated screening protocol and standard operating procedures. This was a deviation from previous approaches where screening was conducting according to the specific presenting need, to a suite of standard screening conducted according to age- and sex-dependent risk factors. For example, an adult male could be offered screening related to physical fitness, nutrition, smoking/substance abuse and mental health [10]. And apart from structured screening tools, clients have access in the clinics to self- assessment tools, including blood pressure monitoring, height, weight, and body mass index measurement [11].

The standard screening protocols indicate the point at which clinic staff intervene with regard to, for example, borderline hypertension, impaired glucose tolerance, and overweight or obesity status. Interventions may include nutrition counselling, and physical activity and smoking cessation programmes [12]. There are currently 401 health clinics that offer counselling, nicotine replacement therapy and varinicline and these currently (2012-13) report a quit rate 16.3% [12].

The National Diabetes Registry (NDR) is another initiative to monitor and evaluate NCD management within the primary care setting. The NDR monitors the primary health care target achievements with regard to clinical investigation results, drug use, complications, and co-morbidities of patients with diabetes. Since 2009, all patients receiving diabetes care at 644 participating health clinics are registered in the NDR, with their status regularly updated.

The integration of NCD management in primary care has increased the role of nurses and medical assistants. This cadre of the health workforce represents a more stable group of health care providers, compared to medical officers who show a high turn-over rate in primary health care settings. Nurses and medical assistants frequently remain in health clinics for several years at each posting, allowing for continuity of care for clients. These health care providers are also well trained to manage the patients with chronic conditions, through for example, diabetes education, clinical care, teaching self-care principles and skills in insulin initiation and titration, as well as training fellow colleagues.

## The NCD Prevention- 1Malaysia (NCDP-1M)

### Background and funding

The NCDP-1M is the main programme under Strategy 1 of the National Strategic Plan for Non-Communicable Diseases (NSP-NCD) 2010-2014, i.e., Prevention and Promotion. It also helps to address Strategy 4 of the Strategic Plan, which is, Multi-Sectoral Approach, because it engages the community as a partner in NCD management. NCDP-1M undertakes nation-wide NCD risk factor screening and intervention across three settings, i.e., community, workplace and schools. The programme aims to achieve population-wide NCD risk factor screening to detect risk factors at their earliest stage, and uses obesity as the main entry point for NCD risk factor intervention [13].

NCDP-1M was launched in October 2010 with approximately 200 projects throughout the country. These projects were supported by a dedicated fund allocated by the Director General of Health, Malaysia. The number of the projects increased, and by 2012, a programme budget of RM4 million annually was approved. The Malaysian Health Promotion Board (MySihat) is a strong collaborative partner, and has provided support for the implementation of NCDP-1M at several sites. As of 31 December 2012, more than 32,000 clients had been supported at 496 NCDP-1M project sites. In total, 55,000 NCD risk factor screening procedures had been undertaken (between October 2010 and December 2012).

### Planning and implementation

NCDP-1M is a nation-wide programme. However, each State determines the extent of their participation and submit formal funding applications to support their selected projects. To increase accountability, District Officers of Health were made responsible for all projects within the respective State and are required to comply with established process indicators. The projects have received significant support from senior levels within the Ministry of Health, and are operational despite limited human resources and the increased burden to existing services.

To support implementation, the Ministry of Health published a series of NCD Community-based Intervention Training Modules which address six disease risk factors, i.e., physical activity and exercise, healthy eating, weight reduction, smoking cessation, stress management, and alcohol use. The Intervention Training Modules are designed for use by non-health professionals; in this case, volunteers from the community. Where an individual meets the criteria for NCD risk, according to the factors measured, they are invited to participate in a semi-structured intervention programme designed by the Community Health Volunteers, with the technical support from Ministry staff. Intervention activities are integrated into the community activities and culture.

Implementation at the State level was coordinated by the State NCD Epidemiologist – who is usually a public health physician. Advocacy was a crucial component of implementation, including with front-line Ministry staff, within the State health department, and with non-health sector, NGOs, and the community. There was a degree of flexibility in how NCDP-1M projects were implemented, with the State NCD Epidemiologist determining the approach deemed suitable for their State.

One State Health Department adopted a “whole-of-government” approach, securing the endorsement and support from the State government to implement NCDP-1M. This was achieved through alignment of the objectives of NCDP-1M with the State government agenda to improve the quality of life of its community. As part of this quality of life agenda, NCD Teams were established at the district level to initiate, facilitate and monitor community-based interventions, including NCDP-1M. These teams are comprised of staff from the main health clinic in the district including a district NCD epidemiologist, a senior medical officer, nutritionists, health education officers, assistant medical officers, and nurses. The district-level NCD Teams was an approach adopted by several other States.

### Community participation and engagement

The participation of the community, through for example Health Volunteers has been crucial to the success of programmes like NCDP-1M. Prior to programmes relating to the prevention of NCDs, the Ministry relied on the support of the community for communicable disease prevention efforts. The willingness of the rural community, in particular, to work with clinic health staff, was key to the success of several early programmes involving the Village Health Promoter ( in Sarawak), the Primary Health Care Volunteer (in Sabah), and the Health Clinic Advisory Panels (nation-wide).

The existence of NCDP-1M is dependent on Health Care Volunteers from the community. These volunteers are nominated by the community itself and are trained in the basic principles of healthy living, pathophysiology, and the epidemiology of NCDs. Apart from educating and advocating for healthy lifestyles, they are also trained to conduct blood sugar tests, body mass index and blood pressure measurements, and to read and interpret results from these screening tests. They are relied upon to institute the established referral mechanism which ensures that clients are provided access to their nearest health clinics for follow-up assessment.

While Health Care Volunteers do not receive monetary rewards, they are formally recognised and acknowledged by the Ministry with certificates of appreciation signed by the Chief Minister, special badges, and sponsored trips to present the successful implementation of the NCDP-1M projects at local scientific meetings. The annual NCDP-1M Convention also provides a forum for recognising successful projects, sharing experiences, and facilitating national networking for Health Care Volunteers.

Community inputs are further solicited through the Health Clinic Advisory Panels. Health Clinic Advisory Panels were initiated in 1995, and consist of local community leaders or people considered to be influential in the community, and healthcare staff from the respective local clinic. There are currently 9,883 registered members of Health Clinic Advisory Panels, in 745 health clinics nationally. The main role of these Advisory Panels is health promotion and prevention advocacy, and providing a ‘bridge’ between the local community and the health clinic.

With approval from the local community, each Panel focusses on different health concerns, for example, NCDs, communicable diseases, or maternal and child health. Examples of activities facilitated by Health Clinic Advisory Panels include community health screening, health camps, health care initiatives for the elderly, and control of dengue and malaria. Community Panel members were also involved in the development of the training modules for NCDP-1M, with the Ministry providing seed money annually for such activities.

Community Health Care Volunteers and community members of the Health Clinic Advisory Panels provide a local community perspective on how to address local health issues. This perspective is valued by and essential to the Ministry, given effective implementation of health interventions, particular when those interventions relate to chronic conditions, are unlikely to be successful without community support.

### Monitoring and evaluation of NCDP-1M

Data from each district is collected through online templates. This online process enables up-to-date monitoring of the various projects. Community-based weight loss programmes for instance provide data on key performance indicators like percentage of clients with weight loss after 6-month follow-up. Paper-based records are maintained for expenditure, training conducted, and membership of District NCD Teams.

Given the issues of hardware availability and internet access, especially in rural and remote regions, it was important for the Ministry of Health to make use of recent and ongoing government efforts to increase internet and computing infrastructure to the rural districts. Negotiations during the implementation of NCDP-1M, with various government sectors and partners, resulted in access to newly available facilities. The success of such negotiations was based on the premise that NCDP-1M provided added value to these information and communication technology (ICT) investments by helping to increase ICT literacy and provide local business opportunities to small and medium-based enterprises.

Another factor attributed to the successful implementation of NCDP-1M was that the Ministry’s Deputy Director General of Health (Public Health) has the main programme outcomes as his personal key performance indicators. This has ensured that continuous support and due attention at the highest levels of the Ministry, are given to the programme. Summaries of data from NCDP-1M are also relayed to the relevant community, to provide incentive, and to unsure sustainability through continuous commitment by the community.

For all that has been effective in the implementation of interventions to prevent and treat NCDs within the primary health care system in Malaysia, there are some limitations. The quality of the projects and implementation teams is not consistent across districts: some centres are understaffed; others have limited resources for other areas of health need and need to make different choices about resources allocation.

## Discussion

The demographic and epidemiological transition present challenges for primary health care as the first point of contact where people seek their medical treatment. NCDs such as diabetes, hypertension and heart disease are complex to manage and because of their chronicity, require concerted efforts from all components of the health system for activities at the population level as well as at the individual level. At the primary care level, the processes in the clinic, manpower, training, equipment, drugs, funding and resource allocation mechanisms facilitate in the management of NCDs. As Malaysia has various types of health clinics with differing capacities and capabilities, standards of care are variable depending on the scope of services and type of resources available. There are still deficiencies in carrying out interventions and prevention of care in some primary care clinics especially the smaller clinics. There are still insufficient diabetes educators and allied health workers in the community, especially the dieticians and the nutritionists.

NCDs can be prevented by managing the risk factors early, as well as early detection of the disease. Unfortunately, since individuals with NCD risk factors, or even at the early stage of NCDs are free from any symptoms (i.e. asymptomatic), they feel healthy and therefore do not access our healthcare services. People who feel well do not want to come to health clinics. Therefore, we need a paradigm shift. We already have the necessary skills and tools available in our health clinics; the next question is how do we transfer these skills and tools to the community level. We cannot transfer our doctors, nurses and assistant medical officers to work at the community level, outside of the health clinics or hospitals since they are already providing essential services to an increasing number of clients accessing our healthcare services. Therefore, we need to identify “new” agents of change and through NCDP-1M and subsequent community-based interventions we hope to create more and more agents of change to enable us to adopt the “whole-of-community” approach more effectively.

The NCD-1M is an important initiative and the progress on implementation to date is an important indication of political will to address the growing NCDs epidemic within the context of the health system. The programme has been designed and is being implemented to respond to the needs of various districts and of the communities within those districts. However there are clearly ongoing challenges. Although the implementation of the programme has led to a system of multi-tasking among the health workers, the projects run as vertical programs within a broader system. This makes it difficult to assess effectively the value added to other sectors of the health system or indeed the contribution of other sectors to NCD control. Furthermore, monitoring of the programme is currently based on NCD indicators of individual participants within communities. While this is consistent with the broader aims of NCDP-1M, as a major strategy, there needs to be further developments that begin to address sustainability within a broader health systems approach [14,15].

Another issue that will need to be addressed is the financial sustainability of the NCDP-1M programme. For instance, there are significant cost implications for extended screening programmes. While the cost may be justified by multiple benefits, this has yet to be ascertained. As a long term strategy therefore, there needs to be systematic compilation of the evidence on which decisions and protocols need to be based [16-18].

Finally, the programme relies predominantly on access to and opportunities for people visiting the health services. As a preventive and health promotion strategy, therefore, it does not reach those who may not have a need to or who for various reasons, do not have access to health services. The extent of this challenge will still need to be assessed in order for solutions to be explored.

## Competing interests

All of the authors have no competing interests to declare.

## Declarations

The cost of funding for this article has been covered by the authors.

This article has been published as part of *BMC Public Health* Volume 14 Supplement 2, 2014: Responsive health systems: working with the community on control of non-communicable diseases (NCDs). The full contents of the supplement are available online at http://www.biomedcentral.com/bmcpublichealth/supplements/14/S2.
